# Generation of 4-vinylguaiacol through a novel high-affinity ferulic acid decarboxylase to obtain smoke flavours without carcinogenic contaminants

**DOI:** 10.1371/journal.pone.0244290

**Published:** 2020-12-21

**Authors:** Thorben Detering, Katharina Mundry, Ralf G. Berger

**Affiliations:** Institut of Food Chemistry, Gottfried Wilhelm Leibniz Universität Hannover, Hanover, Lower Saxony, Germany; Cairo University, EGYPT

## Abstract

Traditional smoke flavours bear the risk of containing a multitude of contaminating carcinogenic side-products. Enzymatic decarboxylation of ferulic acid released from agro-industrial side-streams by ferulic acid esterases (FAE) enables the sustainable generation of pure, food grade 4-vinylguaiacol (4-VG), the impact compound of smoke flavour. The first basidiomycetous ferulic acid decarboxylase (FAD) was isolated from *Schizophyllum commune* (ScoFAD) and heterologously produced by *Komagataella phaffii*. It showed a molecular mass of 21 kDa, catalytic optima at pH 5.5 and 35°C, and a sequence identity of 63.6% to its next relative, a FAD from the ascomycete *Cordyceps farinosa*. The ScoFAD exhibited a high affinity to its only known substrate ferulic acid (FA) of 0.16 mmol L^-1^ and a turnover number of 750 s^-1^. The resulting catalytic efficiency k_cat_ K_M_^-1^ of 4,779 L s^-1^ mmol^-1^ exceeded the next best known enzyme by more than a factor of 50. Immobilised on AminoLink Plus Agarose, ScoFAD maintained its activity for several days. The combination with FAEs and agro-industrial side-streams paves the way for a new generation of sustainable, clean, and safe smoke flavours.

## Introduction

The growing customer demand for natural flavours goes hand in hand with new enzymatic tools providing an alternative access to flavour compounds, which were formerly synthesised from crude oil by often harsh methods. Basidiomycetes, one of the two main divisions of higher fungi, offer a great and almost untouched diversity of biocatalysts. Preferably growing on wood, one of the most recalcitrant natural materials, xylophilic fungi feature potent oxidoreductases and hydrolases, such as laccases and phenolic acid esterases [1, 2]. In line with this, many basidiomycetes produce various and sometimes unique aroma compounds. Besides the well-known champignon flavour impact compound 1-octen-3-ol, they are also capable of producing many other and sometimes rather unusual flavours, such as the 5,7,9-decatrienones with pineapple notes and (+)-nootkatone with grapefruit notes [3, 4].

4-VG, the impact flavour compound of smoke flavour, is produced *via* decarboxylation of FA.

**Fig 1:**
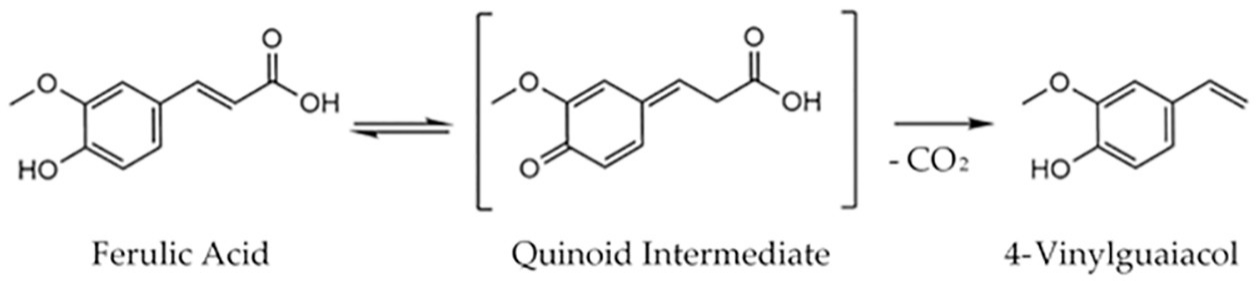
Reaction scheme of the decarboxylation of FA forming 4-VG catalysed by FADs as proposed by Gu *et al*. [8].

Dimers of this phenolic acid crosslink the polysaccharides of plant cell walls, hence increasing their mechanical stability. It is thus present in countless side-products of the food industries, such as cereal brans and sugar beet pulp [5–7]. Its decarboxylation can take place either thermally, as during woodchip pyrolysis, or enzymatically catalysed at mild conditions *via* ferulic acid decarboxylases (FADs) (Fig 1).

Following the mechanism proposed by Gu *et al*., the phenolic hydroxyl group is deprotonated and turned into a quinoid intermediate. The carboxylic group then exits the molecule as carbon dioxide leaving the stable vinylic end product [5].

In contrast to the enzymatic catalysis, the conventional smoking process inevitably generates a number of potent carcinogens including polycyclic aromatic hydrocarbons (PAHs) and heterocyclic amines (HCAs), which may then contaminate smoked foods [9, 10]. Laborious strategies, such as fractionating aroma-rich smoke condensates, may provide healthier products, but require an adequate subsequent disposal of all toxic fractions. Consequently, targeting a new generation of clean, safe and sustainable smoke flavours, new methods are needed producing only the required flavour compounds.

FADs are known from various bacteria, yeasts and further ascomycetes and are divided into three subclasses, namely prFMN dependent, divalent metal dependent and cofactor independent enzymes [11]. The prominent yeast enzyme FDC1 belongs to the class of prFMN dependent decarboxylases and requires a cofactor regenerating system. It shows broader substrate specificities resulting in the formation of the putative carcinogen styrene from cinnamic acid, as it is present in top-fermented beverages, such as wheat beer, and is thus not a suitable candidate [12, 13]. Cofactor independent decarboxylases including bacterial, plant and ascomycetous FADs, require a mandatory hydroxyl group in *para* position of the substrate and are not capable of producing styrene. In contrast to FDC1, the independency from external cofactors turns them into promising candidates for a bioprocess producing 4-VG and its derivatives. However, these enzymes are solely available from heterologous production in *Escherichia coli (E*. *coli)*, a host which is not generally regarded as food-grade. Thus, a potent decarboxylase produced in a safe host is still needed.

This work presents the isolation, biochemical and kinetic characterisation, and classification of a new and highly affine FAD from the basidiomycete *Schizophyllum commune*, its heterologous production in *Komagataella phaffii* and its immobilisation on AminoLink Plus Agarose. The objective was to develop a green chemistry approach as an alternative to conventional smoking techniques, creating a convenient source of pure, natural 4-VG without any unwanted and harmful by-products.

## Materials and methods

### Chemicals, reagents, and substrates

All chemicals, if not marked differently, were purchased from Carl Roth (Karlsruhe, Germany), Merck (Darmstadt, Germany), or Sigma Aldrich (Taufkirchen, Germany). Enzymes were purchased from Thermo Fisher Scientific (St. Leon-Roth, Germany), Nippon Genetics (Dueren, Germany), and New England Biolabs (Ipswich, United States), and oligonucleotides were synthesised by Microsynth Seqlab GmbH (Goettingen, Germany).

### Cultivation of *Schizophyllum commune* and RNA isolation

The basidiomycete *Schizophyllum commune* strain No. 1024 was purchased from the DSMZ (Braunschweig, Germany) and pre-grown for 6 d at 24°C and 150 rpm in SNL media (30.0 g L^-1^ D-(+)-glucose-monohydrate, 4.5 g L^-1^ L-asparagine monohydrate, 3.0 g L^-1^ yeast extract, 1.5 g L^-1^ KH_2_PO_4_, 0.5 g L^-1^ MgSO_4_; 1.0 mL L^-1^ trace element solution (0.08 g L^-1^ FeCl_3_ x 6 H_2_O, 0.09 g L^-1^ ZnSO_4_ x 7 H_2_O, 0.03 g L^-1^ MnSO_4_ x H_2_O, 0.005 g L^-1^ CuSO_4_ x 5 H_2_O, 0.4 g L^-1^ EDTA); pH 6). Inducing the expression of the ScoFAD encoding gene, 25 mL of the pre-culture was added to 250 mL 100 mL L^-1^ SNL containing 10 g L^-1^ wheat bran. The culture was further grown at 24°C and 150 rpm and the intracellular ferulic acid decarboxylase activity was monitored disrupting cells with a homogenisator (Precellys 24, Bertin Technologies SAS, Montigny-le-Bretonneux, France). Mycellium samples were stored overnight in RNAlater solution (Thermo Fisher Scientific, St. Leon-Roth, Germany) and the total RNA was isolated according to the InnuSPEED Bacteria/Fungi RNA Kit (Analytik Jena, Jena, Germany).

### Gene amplification and cloning

The cDNA of the isolated total RNA was synthesised using the FastGene Scriptase II cDNA Kit (Nippon Genetics, Dueren, Germany) and an oligo (dT) primer. Then, the cDNA of the target gene was amplified applying the Phusion High Fidelity Polymerase (Thermo Fisher Scientific, St. Leon-Roth, Germany) with the two specific primers ScoFAD-For (5’- TCGAATATGCCTGGCACTTGG -3’) and ScoFAD-Rev (5’- TCCATCACATCTCTACAGGGTAGG -3’) derived from a genomic sequence (XM_003032814.1) published by Ohm *et al*. [14]. The product was blunt-end ligated to pUC57 linearised with Eco321. The consensus sequence was obtained from five independent positive clones.

### Heterologous expression in *Komagataella phaffii*

The amplified gene was re-amplified to produce N-terminal 10xHis-tagged variants using the primers ScoFAD-For-10xHis (5’-CATCATCATCATCACCATCACCACCACCACCCCGGCACTTGGGAAGAAG-3’) and ScoFAD-Rev2 (5’- TCACAGGGTAGGCCAGCTCAAG-3’) removing the start codon. The product was dephosphorylated by Antarctic Phosphatase (AnP) and blunt-end ligated to pPIC9 cut with *SnaBI* and phosphorylated by T4 Polynucleotide Kinase. The construct linearised by *PmeI* was inserted into the AOX1 locus of competent *K*. *phaffii* GS115 cells via electroporation (Eporator, Eppendorf, Hamburg, Germany) based on a method of Lin-Cereghino *et al*. [15].

The transformed cells were cultivated on histidine-deficient plates and 96 clones were separately cultivated and screened for enzyme activity analogously to Nieter *et al*. [16]. The clone with the highest detectable activity was used for an up-scaled cultivation in 100 mL BMMY media for 4 d at 20°C and 200 rpm after inoculation with 10 mL pre-culture grown for 2 d at 28°C and 200 rpm in YPD.

### Bradford assay

For quantitation of protein concentration, a Bradford-type assay was used (Bio-Rad Protein Assay, Bio-Rad, Hercules, United States). The procedure followed the manual supplied by the manufacturer. The absorption at 595 nm was measured in triplets in a microplate reader (Eon, BioTek, Winooski, United States) against external BSA calibration in the range between 0.1 and 2.0 mg L^-1^.

### Ni-NTA affinity chromatography and SDS-PAGE

The heterologously produced and His-tagged protein was purified via FPLC-based Ni-chelate affinity chromatography (Ni-NTA). Columns were equipped with 10 mL (bed volume) Protino Ni-NTA Agarose (Macherey-Nagel, Dueren, Germany). The chromatography (NGC Quest 10, Bio-Rad, Hercules, United States) with the buffers A (0.5 mmol L^-1^ sodium chloride, 20 mmol L^-1^ Tris, and 20 mmol L^-1^ imidazole) and B (0.5 mmol L^-1^ sodium chloride, 20 mmol L^-1^ Tris, and 500 mmol L^-1^ imidazole) was performed as follows: 16 mL of centrifuged sample (15 min, 15,000 x g) was loaded at a flow rate of 0.25 mL min^-1^, the column was washed 40 mL buffer A, further 40 mL 10 mL L^-1^ B, and 24 mL 50 mL L^-1^ B, and the sample was eluted with 16 mL buffer B followed by washing with 24 mL buffer A. Fractions were collected between 112 and 132 mL of the total volume. The elution fractions were pooled, desalted, and concentrated in spin filters with a cut-off of 3 kDa (VIVASPIN 20, Sartorius, Goettingen, Germany).

### Decarboxylase activity assay and HPLC

At various stages, the decarboxylase activity of samples was quantitated using a generic ferulic acid decarboxylase assay. 25 μL of adequately diluted and centrifuged (15 min, 15,000 xg) sample was mixed with 10 μL Bis-Tris buffer (500 mmol L^-1^, pH 6) and 65 μL substrate solution (3 mmol L^-1^ FA), and incubated at 20°C for 30 min. The reaction was stopped via the addition of 100 μL of acetonitrile. To determine the pH and temperature dependencies of the recombinant enzyme, the buffer was changed to 400 mmol L^-1^ Britton-Robinson buffer (400 mmol L^-1^ H_3_BO_3_, 400 mmol L^-1^ H_3_PO_4_, 400 mmol L^-1^ CH_3_COOH) in the range between pH 2 and 12 and the incubation temperature was set between 5 and 70°C. For the stability experiments, the enzyme solution was pre-incubated at the respective conditions for 60 min.

The samples were analysed using HPLC (LC-10, Shimadzu Deutschland GmbH, Duisburg, Germany) equipped with a reversed phase column (Chromolith Performance RP-18e reverse-phase column, 100 x 4.6 mm, Merck, Darmstadt) and a UV/VIS detector (SPD-10A VP, Shimadzu Deutschland GmbH, Duisburg, Germany). For the separation of FA and 4-VG in 10 μL samples, a stepwise gradient using 1 mL L^-1^ formic acid (A) and acetonitrile (B) as eluents was performed at a total flow rate of 1.5 mL min^-1^: loading the sample in 1.35 mL min^-1^ A and holding for 0.05 min, gradient to 1.05 mL min^-1^ A in 0.1 min, 1.05–0.75 mL min^-1^ A in 1.95 min, 0.75–0.045 mL min^-1^ A in 0.25 min, 0.045–0 mL min^-1^ in 0.95 min, 0–1.275 mL min^-1^ A in 0.4 min, and 1.275–1.35 mL min^-1^ in 1.3 min. The 4-VG concentration was quantitated from the corresponding peak at 263 nm using external calibration. One unit of activity was defined as the amount of enzyme required for converting 1 μmol min^-1^ FA at given conditions. All measurements were obtained from triplets of independent samples. The purity was confirmed by GC-MS as described previously [17].

### Determination of kinetic constants

Determining the affinity and catalytic efficiency parameters, the decarboxylase activity assay was modified applying 0.698 μg of enzyme along with 10 μL of Bis-Tris buffer (500 mmol L^-1^, pH 6) and the following FA concentrations in a regular reaction volume of 100 μL: 33, 65, 98, 130, 163, 195, 228, 260, 293, 325, 390, 455, 520, 585, 650, 813, 975, 1138, 1300, 1463, 1625, 1788, and 1950 μM. The reaction was terminated adding 100 μL of acetonitrile after an incubation for 15 min at 35°C. The conversion rate was calculated from the regular HPLC analysis and K_M_ as well as k_cat_ values were determined by SigmaPlot 12.5 (Systat software) using non-linear regression and the Michaelis-Menten equation.

### Multiple sequence alignment

The obtained sequence of the ScoFAD was aligned against three published phenolic acid decarboxylases using ClustalW (Cornway Institute, UCD, Dublin, Ireland). The parameters were as follows: Gap open penalty: 10.0, gap extension penalty: 0.05, hydrophilic residues: GPSNDQERK, hydrophilic gaps: yes, weight matrix: BLOSUM.

### Immobilisation

The covalent immobilisation was based on a protocol adapted from Siebert *et al*. [18]. Modified spin columns were equipped with a polyethylene filter (30 μm pore diameter) and loaded with 200 μL AminoLink Plus Coupling Resin (Thermo Scientific^™^, Waltham, United States) corresponding to 100 μL bed volume and 4.7 mg dry agarose. The resin was washed by adding 600 μL of coupling buffer (0.1 mol L^-1^ sodium phosphate, 0.15 mol L^−1^ sodium chloride, pH 7.2) and draining via gravity. Equal volumes of the protein sample and coupling buffer were mixed and samples were stored for the calculation of the coupling efficiency. 200 μL of this mixture and 4 μL cyanoborohydride solution (5 mol L^−1^ NaCNBH_3_ in 1 mol L^−1^ NaOH) were added to the column, which was sealed and agitated overnight at 4°C. The flow-through was collected and the coupling efficiency was calculated with 29.8 mg g^-1^ measuring the protein content before and after the coupling reaction by Bradford assay. Further, the resin was washed with 600 μL coupling buffer and 400 μL quenching buffer (1 mol L^-1^ Tris-HCl). Quenching was performed adding 200 μL quenching buffer and 4 μL cyanoborohydride solution prior to agitation at 4°C for 30 min. Two washing steps with 600 μL wash buffer each (1 mol L^−1^ NaCl) were applied, leaving the immobilisate ready for further analysis. To determine the activity of the immobilisate a modified assay, 2.5 mL ddH_2_O, 1 mL buffer (Bis-Tris, 500 mM, pH 6) and 6.5 mL of 3 mM ferulic acid were combined with the sample and regularly incubated. The reaction was stopped adding an equal amount of acetonitrile followed by HPLC analysis. The stability of the enzymatic activity was measured in duplicates in a continuous fixed-bed system pumping substrate (3 mmol L^-1^ FA in 50 mmol L^-1^ Bis-Tris pH 6) through the column at a flow rate of 0.25 mL min^-1^ and quantitating the conversion rate at regular intervals via HPLC.

## Results

### Amplification of the ScoFAD mRNA

To induce the expression of the *ScoFAD* gene, the basidiomycete *Schizophyllum commune* was grown in submerged culture with added wheat bran producing a maximal intracellular enzyme activity of around 5 U L^-1^. The gene target was amplified and a single band was monitored at 500 bp in an agarose gel matching the calculated amplificate length of 516 bp. The construct was Sanger sequenced to confirm the consensus sequence (MT459803).

### Heterologous production and protein purification

**Fig 2:**
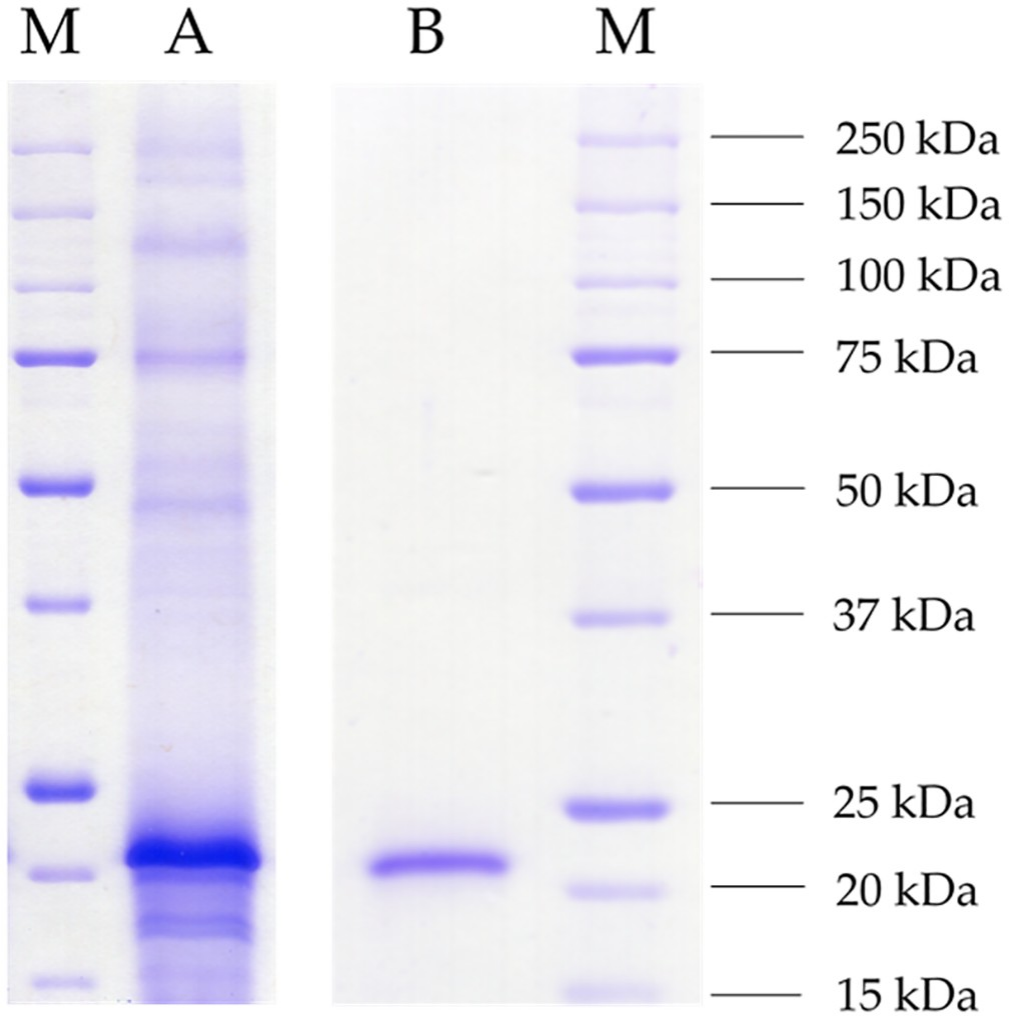
SDS-PAGE (12%) of the raw culture supernatant and the purified enzyme. The culture supernatant of recombinant *Komagataella phaffii* GS115 was harvested at the fourth day of main culture daily induced with 10 mL L^-1^ methanol (A) and the heterologous protein purified via Ni-NTA affinity chromatography (B). As standard protein marker, 5 μL of unstained Precision Protein^™^ (Bio-Rad) was used (M). Both samples were tenfold concentrated using membrane filters (3 kDa cut-off), and 10 μL of each were applied. The gel was stained overnight in Imperial Protein Stain solution (Thermo Scientific) and washed with distilled water.

Competent *Komagataella phaffii* GS115 cells were transformed with an N-terminal His-tagged variant ligated to pPIC9. Following a screening of 96 active clones, an average activity of 2,593 U L^-1^ was obtained from up-scaled cultivation in 100 mL cultures. The culture supernatant showed a distinct band at around 22 kDa matching the calculated monomeric protein mass of 21.05 kDa (Fig 2A). The protein was purified *via* FPLC-based Ni-chelate affinity chromatography. The eluate solely contained a single band of the same protein mass (Fig 2B).

### Biochemical characterisation

**Fig 3:**
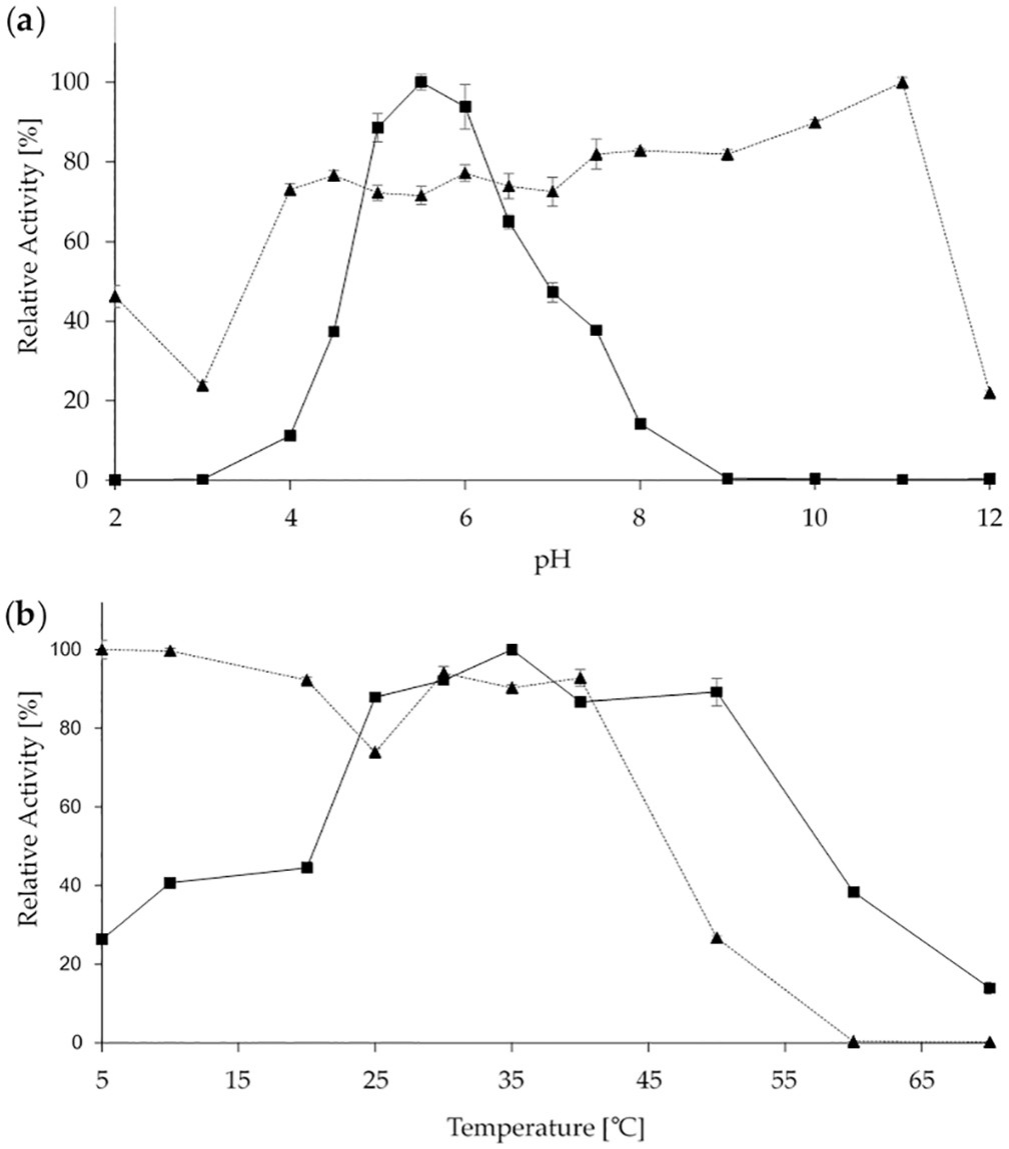
Biochemical characterisation of the heterologously produced ScoFAD. pH (**a**) and temperature (**b**) dependencies of the heterologously produced and purified ScoFAD. Optimum curves are displayed as squares with solid lines and stability curves as triangles and dotted lines. For the pH studies, Britton-Robinson buffer was used. For the temperature stability studies, samples were incubated for one hour at the given temperature prior to the assay. All values were measured in triplicate.

Temperature and pH optima of the purified enzyme were determined (Fig 3). The enzyme showed a pH optimum of 5.5 and detectable activity within the range from 4 to 8. The enzyme was stable in a broad range between pH 4 to 11 exhibiting its highest stability at a strongly alkaline value of 11 (Fig 3a). The ScoFAD showed a broad temperature optimum between 25 and 50°C with a sharp peak at 35°C. The enzyme was stable up to 40°C with no residual activity after incubation for one hour at temperatures above 60°C (Fig 3b).

### Substrate specificity and kinetic characterisation

The substrate specificity was characterised incubating the purified enzyme with cinnamic acid and other phenylpropenoic acids, namely ferulic acid, *p*-coumaric acid, caffeic acid and sinapic acid. The enzyme solely converted FA and was consequently named ScoFAD. This reaction was studied in further detail to determine the affinity and catalytic efficiency and to provide a comparison with other described phenolic acid decarboxylases (Table 1).

**Table 1:** Comparison of various bacterial and fungal phenolic acid decarboxylases.

Enzyme	Accession number	Source	Substrates	K_M_ (mmol L^-1^)	K_cat_ K_M_^-1^ (L s^-1^ mmol^-1^)	Host	T_Opt_ (°C)	pH_Opt_	Reference
IfaPAD	CUI18215	*C*. *farinosa*	*p*CA	0.3	78.4	*E*. *coli*	14	5.5	[17]
			FA	1.9	45.1				
			CA	-	-				
FAD	ACJ26748	*Enterobacter sp*.	FA	2.4	2.1	*E*. *coli*	-	-	[8]
PAD	-	*C*. *guilliermondii*	*p*CA	2.7	42.5	-	25	6.0	[19]
			FA	5.3	21.4				
			CA	-	-				
FDC	AHY75481	*S*. *cerevisiae*	*p*CA	0.11	13.6	*E*. *coli*	35	6.5–7.0	[20, 21]
			FA	0.18	25.5				
			CiA	0.18	21				
AlPAD	LC369499	*A*. *luchuensis*	FA	8.7	87	*E*. *coli*	40	5.7	[22]
			CA	37	3.8				
BaPAD	AKL86192.1	*B*. *atrophaeus*	CA	1.7	-	*E*. *coli*	50	6.0	[23]
			*p*CA	3.5	-				
			FA	2.6	-				
ScoFAD	MT459803	*S*. *commune*	FA	0.16	4779	*K*. *phaffii*	35	5.5	This work

PAD—phenolic acid decarboxylase; FAD/FDC—ferulic acid decarboxylase; *p*CA–*p*-coumaric acid; FA—ferulic acid; CA—caffeic acid; CiA—cinnamic acid and various derivates.

^1^ Substrates are ordered by their respective affinity.

The ScoFAD exhibited an affinity of 0.16 mmol L^-1^ to its only known substrate FA. The turnover number was determined as 750 s^-1^, which resulted in a total catalytic efficiency k_cat_ K_M_^-1^ of around 4,779 L s^-1^ mmol^-1^ (S1 Fig).

### Protein modelling

**Fig 4:**
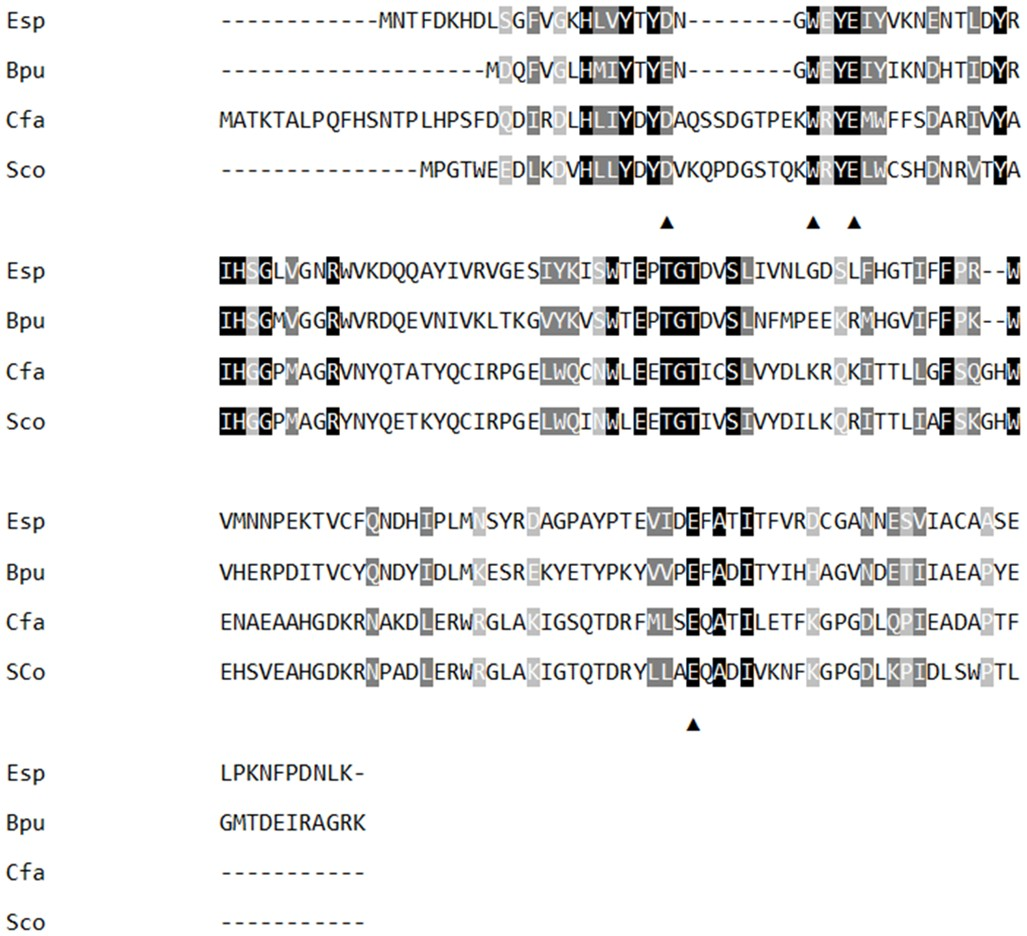
Multiple sequence alignment (MSA) of different phenolic acid decarboxylases. The new ferulic acid decarboxylase ScoFAD described in this work (Sco, MT459803), an ascomycetous *p*-coumaric acid decarboxylase from Cordyceps farinosa described by Linke *et al*. [17] (Cfa, CUI18215), and two bacterial ferulic acid decarboxylases from *Enterobacter sp*. studied by Gu *et al*. [8] (Esp, ACJ26748) and from *Bacillus pumilus* by Matte *et al*. [24] (Bpu, Q45361) were aligned by Clustal W. Conserved residues are marked in black, conservative mutations in dark-grey and semi-conservative mutations in light-grey. The active-site residues proposed by Gu *et al*. [8] are indicated by black triangles.

The protein sequence of the ScoFAD was aligned to the first phenolic acid decarboxylase from a filamentous ascomycete recently described by Linke *et al*. [17] and an enterobacterial FAD described by Gu *et al*. [8] (Fig 4).

**Fig 5:**
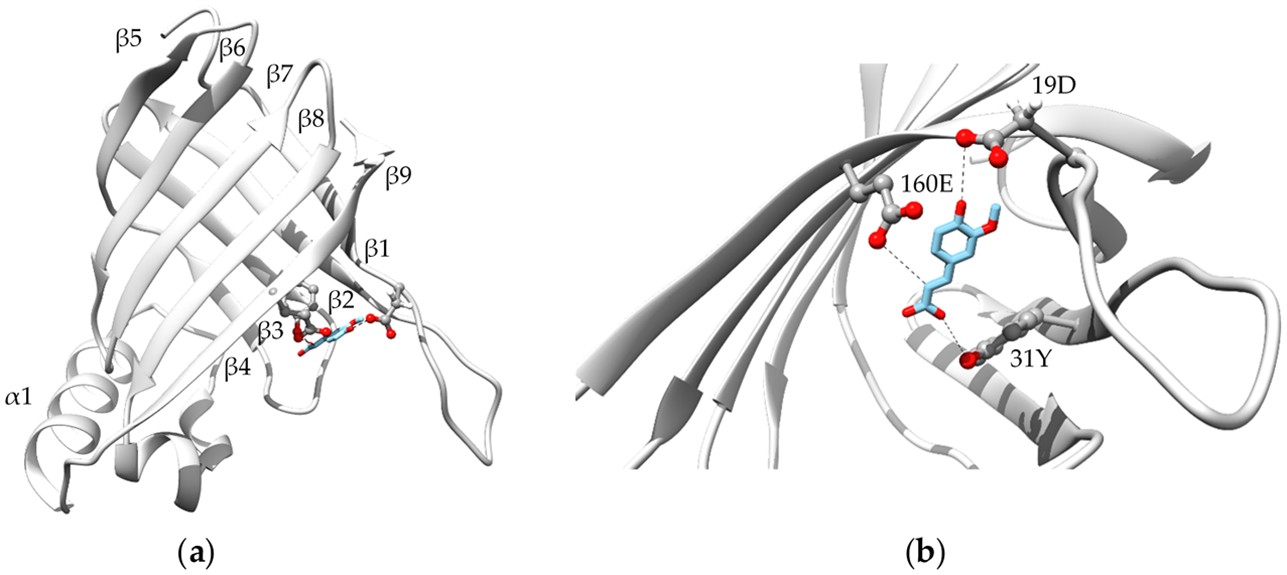
Protein modelling. Overall (**a**) and active-site-focussed (**b**) protein model of the ScoFAD (MT459803) generated with SWISS-MODEL [25] using the template of a phenolic acid decarboxylase from *Bacillus pumilus* (3nad.1) described by Matte *et al*. [24]. The substrate FA was docked according to Gu *et al*. [18] and shown in blue sticks, the active-site residues are shown in stick and balls. The figures were created using UCSF Chimera 1.14.

The ScoFAD (Sco) exhibited a sequence identity of 19.4% to the FAD from *Enterobacter sp*. (Esp), 22.0% to the PAD of *Bacillus pumilus* (Bpu) and of 64.2% to the PAD of *Cordyceps* (Cfa). Following the study by Gu *et al*. [8], the residues being proposedly involved in the catalytic cycle were both found in both the ScoFAD and in the PAD from *Cordyceps*. Consequently, the amino acids 19D, 31Y, and 160E were used for the substrate docking studies, whereas 29W was intentionally left out as it is merely held responsible for the entering and orientation of the substrate (Fig 5). The enzyme structure was characterised by two sets of anti-parallel *β*-sheets and an easily accessible peripheral active site. The FA molecule, fixed by short hydrogen bonds on both ends, fills the space between the glutamyl, aspartyl and tyrosyl side chains of the model.

### Enzyme immobilisation

Enzymatic decarboxylation of FA derived from side-streams of the food industries offers a cheap and sustainable pathway to 4-VG at mild reaction conditions. The product may be used in clean and safe smoke flavours. Immobilisation is a common tool to establish a bio-economic process, as less enzyme is needed. Depending on the applied technique, the products are more or less enzyme free and the catalysts can be re-used for subsequent reaction cycles. Especially covalent immobilisation suits these requirements, as in adsorptive techniques, although being more straightforward, enzyme leaking can be seen as was recently reported for a basidiomycetous chlorogenase by Siebert *et al*. [18]. Hence, a covalent immobilisation strategy was used for the ScoFAD involving the formation of a Schiff base between secondary amines of the protein and the aldehyde functionalised resin (Table 2).

**Table 2:** Activity data for the immobilisation of ScoFAD to AminoLink Agarose.

Sample	Activity [U mL^-1^]	Specific activity [U mg^-1^ protein]	Immobilisate activity [U mg^-1^ resin]	Absolute activity	Relative Activity [%]
Pre-immobilisation	3.62 ± 0.1	2.92 ± 0.1	-	0.72 ± 0.02	100
Post-immobilisation	0.0072 ± 0.001	n.d.	-	0.014 ± 0.0001	2
Immobilisate	-	2.15 ± 0.04	0.13 ± 0.002	0.57 ± 0.009	78

n.d.—not detected

Comparing the unbound and the immobilised enzyme, a drop in specific activity from 2.92 U/mg to 2.15 U/mg was monitored. However, 78% of the initial activity was recovered in the immobilisate.

**Fig 6:**
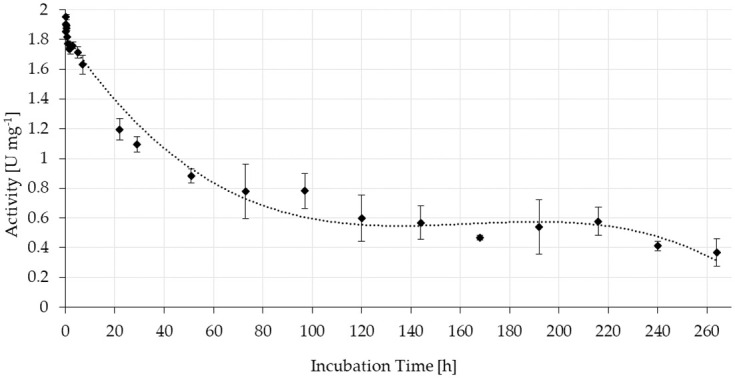
Stability of the specific activity of ScoFAD covalently immobilised to AminoLink Plus Agarose. In duplicate measurement, 0.28 mg of purified enzyme were bound to 4.7 mg of resin, which corresponded to a bed volume of 100 μL. Substrate solution was pumped through the columns at room temperature with a flow rate of 0.25 mL min^-1^ for 264 h while collecting samples of the flow through mixing it with the same volume of acetonitrile. The concentration of 4-VG was quantitated using UV-Vis-HPLC and external calibration.

Consequently, stability tests with FA in buffered solution were monitored over 264 h of continuous reaction using columns filled with the solid resin (Fig 6).

During the first 48 h of operation, the activity declined to about half the initial value. It then stabilised at around 0.6 U mg^-1^ resin corresponding to around 40% of the initial activity and remained around this level until 216 h. The experiment was ended after 264 h (11 d) of running at a level of 0.37 U mg^-1^ (around 20%). At this point, roughly 4 L of substrate solution had passed the column. This corresponded to a total of a 40,000 fold of the bed volume of 100 μL.

## Discussion

In this work, the first basidiomycetous phenolic acid decarboxylase was studied. The closest relatives producing analogous enzymes are ascomycetes, the second main class of higher fungi. Examples are the well-studied FDC1 from *Saccharomyces cerevisiae*, the AlPAD from *Aspergillus luchuensis* and the recently described IfaPAD from *Cordyceps farinosa* [17, 22, 26, 27].

For the first time, a phenolic acid decarboxylase was produced heterologously in a yeast. The activity in the *Komagataella* culture exceeded the activity in the basidiomycetous culture by more than a factor of 500. In contrast to the common host *E*. *coli*, *Komagataella phaffi* is classified as safe (GRAS) by the United States Food and Drug Administration (FDA) [28, 29]. Other decarboxylases including the BaPAD were usually produced in *E*. *coli* [23]. Further, the recombinant ScoFAD was secreted into the medium, which significantly simplified downstream processing.

In contrast to other characterised enzymes, the ScoFAD showed an unusually broad temperature optimum between 25 and 50 degrees C enabling a wide range of possible process temperatures at only little activity loss [17, 19]. Likewise, the enzyme remained highly stable against temperatures up to 40°C, which provides flexible incubation conditions. With a pH optimum of 5.5, it closely matched other cofactor-independent decarboxylases from bacterial and fungal sources, which use acid catalysis instead [17, 22, 23]. Similar optima were detected for many more basidiomycetous enzymes including a ferulic acid esterase from *Rhizoctonia solani* [1]. Both enzymes may thus be promising candidates to work hand-in-hand releasing and converting ferulic acid from lignified natural sources in a two-step enzymatic process.

In spite of the rather low overall identity of 19.4%, MSA analysis suggested the four conserved residues 19D, 31Y, 160E, and 29W in the active site, which were previously stated to be involved in the catalytic cycle of the FAD from *Enterobacter sp*. [8]. Despite being based on the crystal structure of a PAD from *Bacillus pumilus*, the localisation of these residues matched the binding of FA in a protein model [24].

In contrast, the ScoFAD offered a high identity of 64.2% to the recently published IfaPAD [17]. However, FA was the only known substrate of the ScoFAD, whereas the IfaPAD also converted *p*-CA. The newly identified enzyme actually exhibited by far the highest known affinity to FA with a K_M_ value of 0.16 mM. In contrast, affinities for FA of other fungal decarboxylases typically ranged between 0.8 and 8.7 mM [22, 26]. As ScoFAD accepts only FA as a substrate, the enzyme may be used on complex plant materials without the risk of generating unwanted side-products, particularly the putative carcinogen styrene from cinnamic acid. Based on the also relatively high turnover number (k_cat_ of 750 s^-1^), the resulting catalytic efficiency (k_cat_/K_M_ of 4,779 L s^-1^ mmol^-1^) exceeded the second highest by more than a factor of 50 [18].

As the ScoFAD required a hydroxyl group in *para* position, it did not require any external cofactors and showed a close relationship to further enzymes of this class in MSA, protein modelling and biochemical properties, the enzyme can be clearly classified as cofactor-independent phenolic acid decarboxylase [11].

ScoFAD is the first successfully immobilised enzyme for the production of 4-VG in a fixed bed approach. Around 80% of the applied activity was found in the immobilisate demonstrating the suitability of the immobilisation method for a feasible bioprocess. An analogous strategy was recently applied for the removal of chlorogenic acid from coffee beverages [18]. After 264 h and 40,000 passed bed volumes a remaining activity of 20% was measured. The only further example of an immobilised PAD was recently published by Mittmann *et al*. [30], who demonstrated a working and stable all-enzyme hydrogel for the production of *p*-hydroxystyrene, another smoke flavour compound. The immobilisate may be used for the continuous production of 4-VG from FA released from side-streams of the food industries by ferulic acid esterases as shown by Nieter *et al*. [16].

## Conclusions

Many basidiomycetous oxidoreductases, mainly laccases, arylalcohol oxidases, and certain peroxidases, all involved in the degradation of lignocellulose, have been studied in great detail. Accessory enzymes such as phenolic acid decarboxylases have received much less attention. The reported ScoFAD offers an example of the uniqueness and capability basidiomycetous enzymes often exhibit. Its catalytic efficiency exceeds all other FADs published so far. The high activities generated by the food-grade expression host *Komagataella phaffii* GS115 and the successful immobilisation pave the way for a future sustainable production of natural 4-VG for clean and safe smoke flavours. At the same time, the findings should stimulate the research for other unique enzymes from basidiomycetes, particularly those revolving around phenol biochemistry. To improve the thermal and operational long-term activity of the immobilisates, other carrier materials and linker chemistries can be envisaged.

## Supporting information

S1 Table List of chemicals. (DOCX)

S1 Fig Michaelis-Menten non-linear-regression for the conversion of ferulic acid by ScoFAD. (TIF)

S1 Raw images Raw SDS-PAGE images. The images were obtained using Epson Perfection V39 scanner at 24 bit and 1200 dpi without any correction algorithms. Panels “A”, “B”, and “M” were used in Fig 2 under the same label, whereas panels “X” were not used.
(PDF)
