# Supplementary material for: Generation of 4-vinylguaiacol through a novel high-affinity ferulic acid decarboxylase to obtain smoke flavours without carcinogenic contaminants
Source: PLoS One. 2020 Dec 21;15(12):e0244290. doi: 10.1371/journal.pone.0244290 (PMC7751879; doi:10.1371/journal.pone.0244290)
Supplement: S1 Table — (DOCX) [file pone.0244290.s001.docx]

S1 Table. List of chemicals.

|  | | **Chemical** | **Purity** |  | **Source** |  |  |
| --- | --- | --- | --- | --- | --- | --- | --- |
| **Media** | | Agar-Agar, Kobe I |  | | Carl Roth |  |  |
|  | | L-Asparagine monohydrate | ≥99 %, Ph.Eur. | | Carl Roth |  |  |
|  | | Biotin | ≥99 % | | Sigma Aldrich |  |  |
|  | | Copper(II) sulfate pentahydrate | Puriss., p.a. | | Honeywell |  |  |
|  | | Ethylendiamine-tetraaceticacid disodium salt dihydrate | ≥99 %, p.a., ACS | | Carl Roth |  |  |
|  | | Ferric chloride Hexahydrate | Puriss., p.a., ACS | | Honeywell |  |  |
|  | | D-(+)-Glucose monohydrate |  | | Merck |  |  |
|  | | Magnesium sulfate | ≥99 %, p.a., water-free | | Carl Roth |  |  |
|  | | Peptone ex casein |  | | Carl Roth |  |  |
|  | | Potassium dihydrogen phosphate | ≥98 %, Ph.Eur., BP | | Carl Roth |  |  |
|  | | di-Potassium hydrogen phosphate | ≥99 %, p.a., water-free | | Carl Roth |  |  |
|  | | Yeast extract |  | | Merck |  |  |
|  | | Yeast nitrogen base |  | | Sigma Aldrich |  |  |
|  | | Zinc sulfate heptahydrate | P.a. | | Merck |  |  |
| **Buffers** | | Acetic acid | 100 %, p.a. | | Carl Roth |  |  |
| **and eluents** | | Acetonitrile | ≥99.9 %, gradient grade | | Honeywell |  |  |
|  | | Bis-(2-hydroxy-ethyl)-amino-tris(hydroxymethyl)-methane | ≥99 % | | Carl Roth |  |  |
|  | | Boric acid | ≥99.5 %, ACS | | Sigma Aldrich |  |  |
|  | | Ethanol | ≥96 % | | VWR |  |  |
|  | | Formic acid | ≥99 %, p.a., ACS | | Carl Roth |  |  |
|  | | Hydrochloric acid 32 % | p.a., ISO | | Carl Roth |  |  |
|  | | Imidazole | ≥99 % | | Carl Roth |  |  |
|  | | Methanol | Gradient grade | | VWR |  |  |
|  | | *o*-Phosphoric acid | 85 %, Ph.Eur. | | Carl Roth |  |  |
|  | | Potassium hydroxide | ≥85 % | | Carl Roth |  |  |
|  | | Sodium chloride | ≥99.8 %, with anti caking agent | | Carl Roth |  |  |
|  | | di-Sodium dihydrogen phosphate dihydrate | ≥98 %, p.a., ACS | | Carl Roth |  |  |
|  | | Sodium dihydrogen phosphate monohydrate | ≥98 %, p.a., ACS | | Carl Roth |  |  |
|  | | Sodium hydroxide | ≥98 %, p.a., ISO | | Carl Roth |  |  |
|  | | Tris(hydroxymethyl)aminomethane | ≥99.3 % | | Carl Roth |  |  |
| **Standards** | | Caffeic acid | ≥98 % | | Carl Roth |  |  |
|  | | Cinnamic acid | ≥99 % | | Honeywell |  |  |
|  | | *p-*Cumaric acid | ≥98 % | | Carl Roth |  |  |
|  | | *trans*-Ferulic acid | 99% | | Sigma Aldrich |  |  |
|  | | Sinapic acid | ≥98 % | | Sigma Aldrich |  |  |
|  | | 4-Vinylguaiacol |  | | Fluorochem |  |  |
| **SDS-PAGE** | | Rotiphorese Gel 40 (37.5:1) |  | | Carl Roth |  |  |
|  | | Sodium dodecyl sulfate | ≥99 % | | Carl Roth |  |  |
|  | | Bromophenol Blue | ACS | | Merck |  |  |
|  | | Ammonium persulfate | >98 %, p.a., ACS | | Carl Roth |  |  |
|  | | N,N,N′,N′-Tetramethylethylenediamine |  | | VWR |  |  |
|  | | 1.4-Diothiothreitol | >99 %, p.a. | | Carl Roth |  |  |
|  | | Imperial Protein Stain |  | | Thermo Scientific |  |  |
|  | | Glycerol | ≥99.5 %, p.a. | | Carl Roth |  |  |
| **Immobilisation** | | Sodium cyanoborohydride | 95 %, reagent grade | | Sigma Aldrich |  |  |
|  | | AminoLink Plus Coupling Resin |  | | Thermo Scientific |  |  |
| **Misc** | | BSA | ≥96 % | | Sigma Aldrich |  |  |
|  | | RNAlater solution |  | | Thermo Scientific |  |  |
|  | | D-Sorbitol | ≥98 % | | Sigma Aldrich |  |  |
|  | | Bradford Reagent for 0.1-1.4 mg ml^-1^ protein |  | | Sigma Aldrich |  |  |
|  | Fluorochem, Hadfield, United Kingdom; Honeywell, Charlotte, United States; Merck, Darmstadt, Germany; Carl Roth, Karlsruhe, Germany; Sigma Aldrich, St. Louis, United States; Thermo Scientific, St. Leon-Roth, Germany; VWR, Radnor, United States. | | | | | | |
