# Supplementary figures and images for: Generation of 4-vinylguaiacol through a novel high-affinity ferulic acid decarboxylase to obtain smoke flavours without carcinogenic contaminants
Source: PLoS One. 2020 Dec 21;15(12):e0244290. doi: 10.1371/journal.pone.0244290 (PMC7751879; doi:10.1371/journal.pone.0244290)

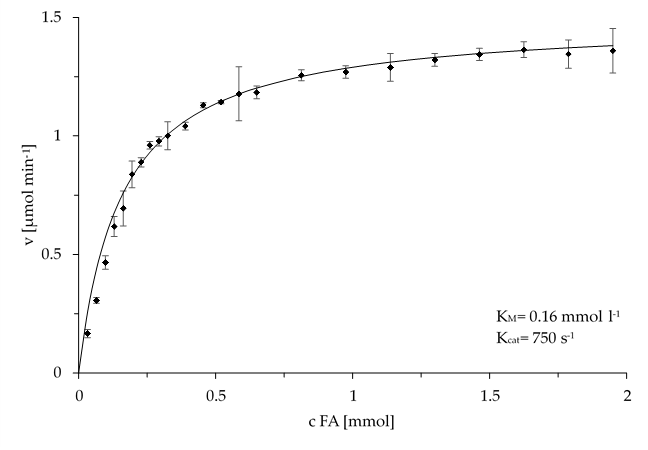

Supplement: S1 Fig — (TIF) [file pone.0244290.s002.tif]

X X A M X X X X X X

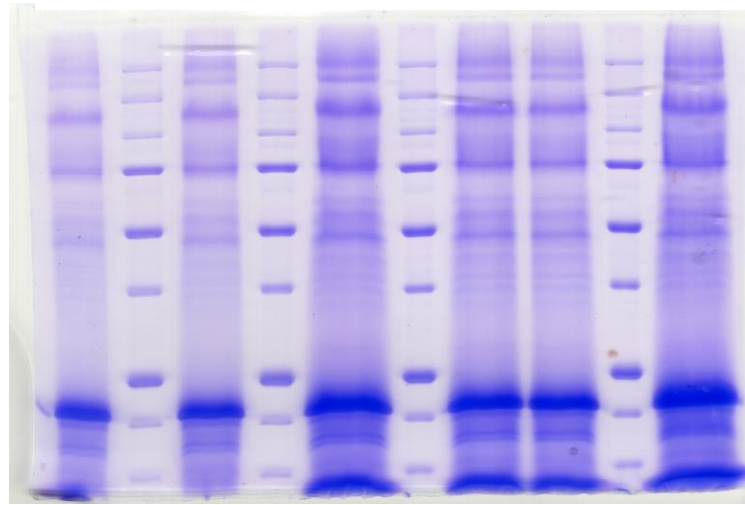

B M X X X X X X X

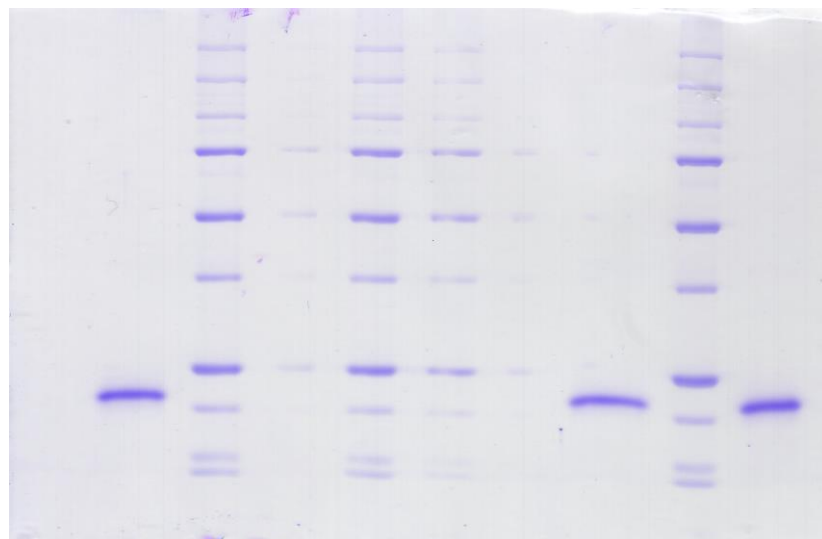

Supplement: S1 Raw images — The images were obtained using Epson Perfection V39 scanner at 24 bit and 1200 dpi without any correction algorithms. Panels “A”, “B”, and “M” were used in Fig 2 under the same label, whereas panels “X” were not used. (PDF) [file pone.0244290.s003.pdf]
